# Multimodal targeted high relaxivity thermosensitive liposome for *in vivo* imaging

**DOI:** 10.1038/srep17220

**Published:** 2015-11-27

**Authors:** Maayke M. P. Kuijten, M. Hannah Degeling, John W. Chen, Gregory Wojtkiewicz, Peter Waterman, Ralph Weissleder, Jamil Azzi, Klaas Nicolay, Bakhos A. Tannous

**Affiliations:** 1 Experimental Therapeutics and Molecular Imaging Laboratory, Neuroscience Center, Massachusetts General Hospital, Boston, MA 02114 USA; 2 Division of Neuroradiology, Department of Radiology, Massachusetts General Hospital, Boston, MA 02114 USA; 3 Center for Systems Biology, Massachusetts General Hospital, Boston, MA 02114 USA; 4 Program in Neuroscience, Harvard Medical School, Boston, MA 02114 USA; 5 Department of Biomedical Engineering, Biomedical NMR, Eindhoven University of Technology, Eindhoven, the Netherlands; 6 Department of Neurosurgery, Leiden University Medical Center, Leiden, the Netherlands; 7 Transplantation Research Center, Renal Division, Brigham and Women’s Hospital and Children’s Hospital, Harvard Medical School, Boston, Boston, MA 02114 USA

## Abstract

Liposomes are spherical, self-closed structures formed by lipid bilayers that can encapsulate drugs and/or imaging agents in their hydrophilic core or within their membrane moiety, making them suitable delivery vehicles. We have synthesized a new liposome containing gadolinium-DOTA lipid bilayer, as a targeting multimodal molecular imaging agent for magnetic resonance and optical imaging. We showed that this liposome has a much higher molar relaxivities r1 and r2 compared to a more conventional liposome containing gadolinium-DTPA-BSA lipid. By incorporating both gadolinium and rhodamine in the lipid bilayer as well as biotin on its surface, we used this agent for multimodal imaging and targeting of tumors through the strong biotin-streptavidin interaction. Since this new liposome is thermosensitive, it can be used for ultrasound-mediated drug delivery at specific sites, such as tumors, and can be guided by magnetic resonance imaging.

Magnetic resonance imaging (MRI) is a routine diagnostic tool with many advantages compared to other imaging modalities including its noninvasive character, lack of radiation burden and its excellent spatial and temporal resolution^1^,^2^. Despite its many advantages, there are intrinsic limitations caused by MRI contrast agents, such as short vascular half-life circulation, which could lead to potential side effects. This drawback can be overcome by using liposomes which can incorporate a high payload of gadolinium-containing amphiphilic lipid in their bilayer resulting in a spectacular increase in effective longitudinal (r1) relaxivity per particle^3^,^4^,^5^.

Hydrophilic drugs can potentially be localized in the aqueous compartment while hydrophobic drugs can be incorporated in the lipid bilayers core of liposomes^6^,^7^. These characteristics together with the potential of liposomes to partition into the immune system, target cells, and reduce unwanted systemic side-effects are important characteristics for the use of liposomes in drug delivery^3^,^4^,^8^,^9^. In addition liposomes can be modified and grafted with different targeting moieties including antibodies or bioresponsive components such as biosensitive lipids or polymers^7^. Due to these characteristics, liposomes have been considered as vehicles for imaging agents since they can either encapsulate a contrast agent, a fluorophore for optical imaging, or both within their interior or in their bilayer. Liposomal imaging agents have the potential to overcome problems associated with rapid clearance, non-specific cellular interaction, and toxicity resulting in poor contrast and low resolution images^3^,^7^.

Multifunctional liposomes have been developed for image-guided drug delivery by incorporating both therapeutic drugs and contrast agents for MRI^3^,^4^. Commonly, lipid-based contrast agents incorporate amphiphilic agents such as gadolinium(Gd)-DTPA derivatives within the membrane moiety of the liposome, leaving the lumen to encapsulate therapeutic molecules^1^,^10^. The disadvantage of these liposomes is that they contain a single Gd-chelate moiety per molecule leading to lower MRI sensitivity. In an attempt to overcome these limitations, macromolecular Gd-chelates with several residues in the single molecular chain have been used, however, these polychelates could change the liposome structure, affecting its surface properties^1^,^11^. Thermosensitive liposomes have been developed for ultrasound-mediated drug delivery at specific sites, such as tumors^12^,^13^,^14^, and can be guided by imaging^10^,^15^,^16^. These liposomes typically incorporate a contrast agent within their lumen where relaxivity is low when the liposome is intact and only increases during the gel-to-liquid phase transition of the liposome^17^. Therefore these liposome formulations are unsuitable for imaging at physiological temperatures, which is necessary for all solely diagnostic purposes.

A possible alternative strategy to enhance detectability with MRI is to increase the effective longitudinal relaxivity for each Gd-chelate moiety. Instead of increasing the amount of Gd particles in the moiety, we hypothesized that the longitudinal relaxivity could be enhanced by increasing the effectivity of the liposome as a contrast agent by ensuring the contribution of Gd particles facing the inner core of the liposome to the relaxivity as the Gd particles are linked to the lipids, which are in both sides of the bilayer. During the gel-to-liquid crystalline phase transition of the liposome, the transmembrane permeability towards solutes and water will be increased^18^. Thus, when using lipids with gel-to-liquid crystalline phase transition temperatures below the physiological temperature to fabricate the liposome, which results in the liposome having a gel-to-liquid crystalline phase transition temperature below physiological temperature, will increase the transmembrane permeability to water of the liposome. This increase in transmembrane permeability to water should positively affect the contribution of the Gd particles in the inner side of the liposome to the relaxivity. In this study, we synthesized and characterized a new thermosensitive liposome, named NLP, which contains Gd-DOTA lipid bilayer and has a transition temperature around 42 °C, due to the addition of lipids with transition temperatures below 45 °C. We compared the relaxivity of this liposome to a previously published conventional liposome formulation (CLP; with a transition temperature of 60 °C) containing Gd-DTPA-BSA lipid^19^ and showed that NLP has a much higher efficacy as an MRI contrast agent. By embedding the gadolinium imaging agent in the membrane of the NLP liposome, this agent exhibits high intrinsic relaxivity when the liposome is intact, making it suitable for monitoring the delivery of drug carriers to the target as well as using the liposome formulation as a contrast agent for diagnostic purposes. Further, we engineered a targeting multifunctional liposome, based on NLP, carrying both gadolinium and a fluorophore in the lipid bilayer as well as biotin on its surface, which can be used to target any ligand using a streptavidin linker. We demonstrated as a proof of principle that this liposome could be used for multimodal molecular imaging, using MRI and fluorescence, and could be targeted to tumors through the strong biotin-streptavidin interaction. Due to the biotin label, multiple ligands can be linked via the strong biotin-streptavidin interaction, which makes the liposome suitable for multi-targeted delivery.

## Results

### Synthesis and characterization of the NLP liposome formulation

**Figure 1:**
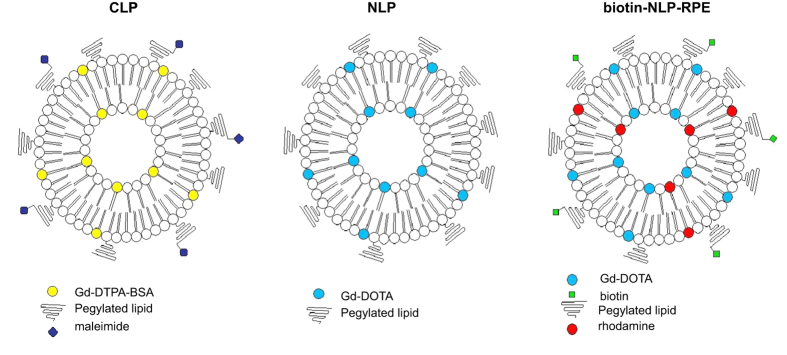
Schematic overview of CLP, NLP and NLP-biotin-RPE liposomes. We synthesized a new DPPC-based liposome (NLP) containing gadolinium-DOTA lipid and compared its characteristics to more conventional DSPC-based liposome with gadolinium-DTPA-BSA (CLP). In order to make NLP multifunctional, a biotin and rhodamine containing lipid was added to the formulation so the liposome can be traced both with magnetic resonance and fluorescence imaging and can be targeted using the strong biotin-streptavidin interaction (NLP-biotin-RPE).

**Figure 2:**
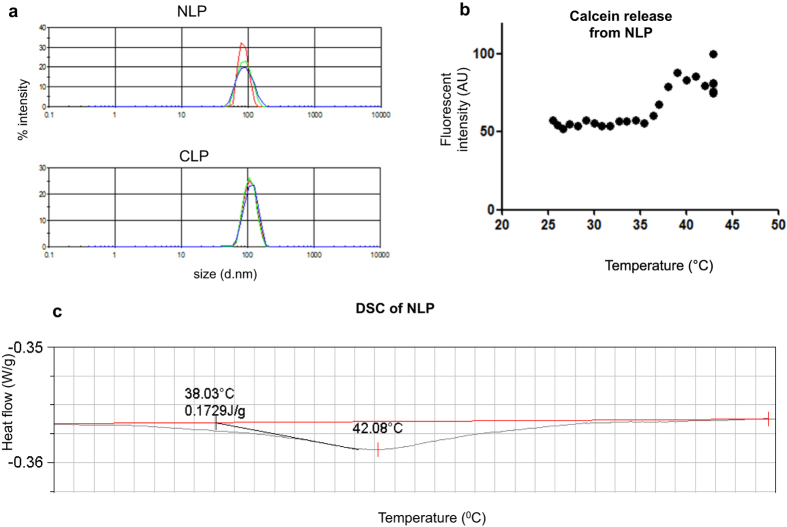
Size distribution and thermosensitivity of NLP and CLP liposomes. (**a**) The size of the liposome was measured with dynamic light scattering. Intensity plots showing the average hydrodynamic diameters of NLP and CLP to be 86 nm and 109 nm respectively. (**b**) NLP-containing calcein was heated at different temperatures. The fluorescent intensity starts to increases around 37–38 °C indicating lipid rearrangement and further increases until around 39 °C. No significant further increase is visible upon addition of the lysis reagent triton X-100. (**c**) Differential scanning calorimetry (DSC) showing that NLP has a transition temperature of 42 °C.

We have synthesized a liposome with gadolinium containing lipids as a new contrast agent for MRI (Fig. 1). We compared the characteristics of this new formulation (NLP) to a more conventional DSPC-based Gd-containing liposome (CLP, Fig. 1; Table 1). We first measured the mean average hydrodynamic diameter of the NLP liposome and showed it to be 86 nm, which is smaller than the conventional CLP liposome having a size of 109 nm (Fig. 2a and Table 2). The mean average diameter of the NLP liposome, stored at 4 °C, was again investigated after 5 weeks. We observed that both the size and size distribution of the liposome remained constant with an average hydrodynamic diameter of 85 nm. The consistency of these results indicates a sterically-stabilized liposome preparation with a shelf lifetime of at least 5 weeks.

In order to determine the efficacy of the different liposomes as contrast agents, we determined the gadolinium ion concentration in both NLP and CLP. The Gd(III) concentration estimated by phosphate determination for CLP and NLP was 2.790 mM and 1.925 mM respectively (Table 2). The Gd(III) concentration in these liposomes was also precisely determined using inductively coupled plasma mass spectroscopy (ICP-MS) and found to be 2.920 mM and 2.238 mM, respectively, similar to the concentration found by phosphate determination (Table 2).

### Relaxivity determination of NLP liposome

The composition of NLP formulation could potentially make this liposome thermosensitive, which could influence both the longitudinal and transversal relaxivities due to a change of water exchange over the membrane. We therefore evaluated whether the NLP is indeed thermosensitive by performing a calcein release experiment during heating. The fluorescent signal of calcein is quenched due to its high concentration (30 mM) inside the aqueous lumen of the liposome. During phase transition, the lipids in the liposome rearrange and the liposome shell opens resulting in calcein release and hence increased fluorescence signal. Upon liposome heating, lipid rearrangement started at 38 °C resulting in leakage of its content (Fig. 2b). The fluorescent signal further increased until around 39 °C at which temperature all calcein appeared to be released. No further increase in fluorescence signal was visible upon the addition of the lysis reagent Triton X-100, showing that all calcein was already released during the 40 minutes heating period. The transition temperature of NLP defined as the temperature at the top of transition phase was determined with differential scanning calorimetry (DSC) and found to be 42 °C (Fig. 2c). These results indicate that NLP is thermosensitive, which could release its contents upon heating.

**Figure 3:**
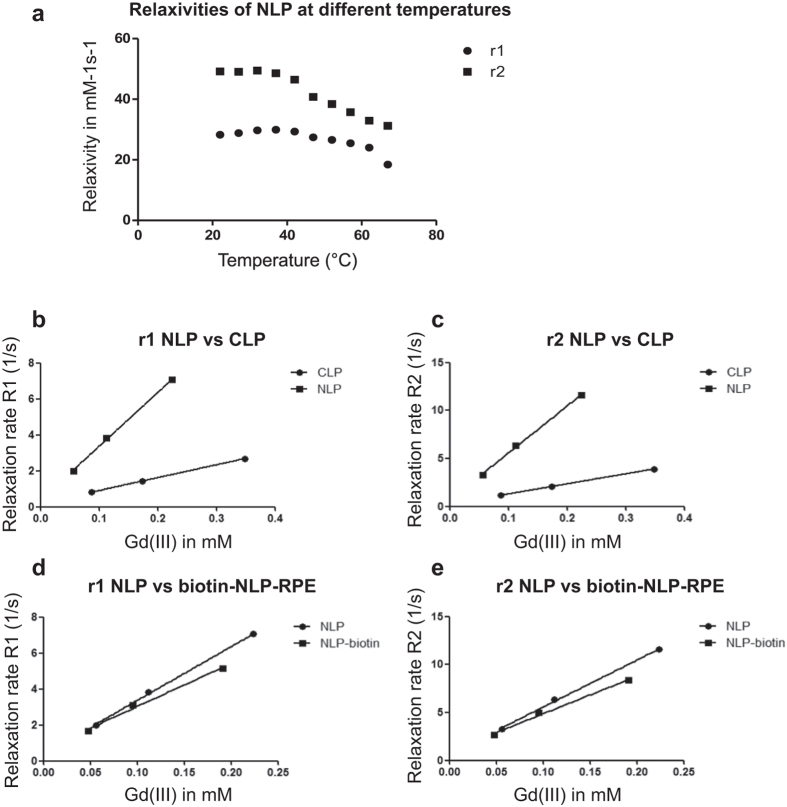
Longitudinal and transversal relaxivities of the different liposome formulations in mM^−1^s^−1^. Longitudinal (r1) and transversal (r2) relaxivities were obtained from NMR measurements at 60 MHz and 1.41 Tesla. (**a**) longitudinal and transversal relaxivities of NLP liposomes obtained at different temperatures ranging from 22 °C to 67 °C with 5 °C intervals. (**b**,**c**) Longitudinal and transversal relaxation rates of NLP as compared to CLP liposomes obtained at 37 °C. (**d**,**e**) Relaxation rates of NLP versus NLP-biotin-RPE are presented.

Further the measured Gd(III) concentration was used to determine the longitudinal (r1) and the transversal (r2) relaxivities of the different liposome formulations. Since we have observed that NLP is thermosensitive (Fig. 2b), r1 and r2 relaxivities were determined at different temperatures to investigate the influence of hyperthermia on the imaging efficacy of this liposome. Longitudinal and transversal relaxivities of the liposome formulations at different temperatures were obtained from the slope of the linear fit of relaxation rates as a function of Gd(III) concentration. At higher temperature, both r1 and r2 relaxivities of the NLP decreased showing a negative influence of hyperthermia on imaging efficacy of NLP (Fig. 3a).

To determine the efficacy of NLP as a contrast agent, r1 and r2 relaxivities at 37 °C were compared to the conventional CLP liposome. T1 and T2 measurements were performed at 60 MHz corresponding to a magnetic field strength of 1.41 Tesla. The Gd(III)DTPA-BSA-based CLP liposome exhibited a longitudinal r1 relaxivity of 7.52 mM^−1^s^−1^ (Table 3, Fig. 3b), which is similar to values published for the same formulation (7.5 mM^−1^s^−1^)^20^. The transversal r2 relaxivity of CLP was found to be 10.6 mM^−1^s^−^1. On the other hand, the Gd(III)DOTA-DSPE based NLP liposome exhibited four-fold higher r1 of 29.9 mM^−1^s^−1^ and five-fold higher r2 of 49.0 mM^−1^s^−1^ as compared to CLP (Table 3, Fig. 3b,c). The longitudinal relaxivity of NLP was >2-fold higher compared to the published relaxivity for DSPC-based Gd(III)DOTA-DSPE containing liposome (12.8 mM^−1^s^−1^)^20^. The reported relaxivities are the average relaxivities of the Gd(III) ions in the inner and outer leaflets of the lipid bilayer.

### Synthesis and characterization of a targeted multimodal NLP liposome

In order to increase sensitivity and selectivity of the newly formulated contrast agent, targeted NLP was synthesized with a biotin on its surface (biotin-NLP). Further, to make NLP suited for multimodal imaging (fluorescence and MRI), rhodamine-PE (RPE) was added within the lipid bilayers of the liposome (biotin-NLP-RPE; Fig. 1). The addition of biotin and rhodamine to the NLP formulation did not affect its size (93 nm, Table 1). The gadolinium ion concentration as well as the longitudinal and transversal relaxivities of biotin-NLP-RPE were found to be similar to the non-targeted NLP liposome (Tables 2 and 3; Fig. 3d,e).

### Targeting of cells with biotin-NLP-RPE in culture

**Figure 4:**
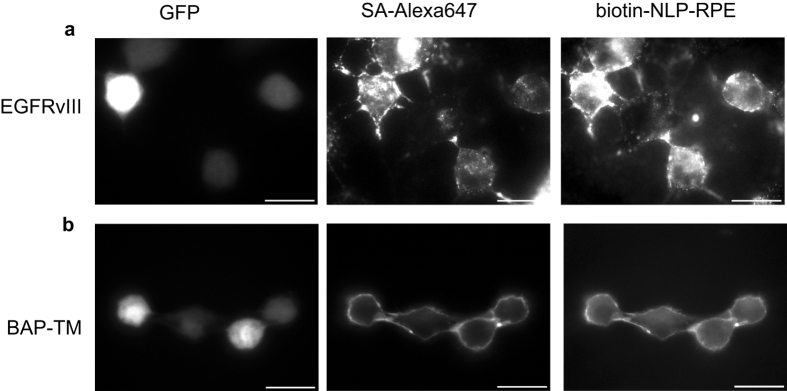
Targeting of cells with biotin-NLP-RPE liposome. (**a**) Gli36 human glioma cells expressing the EGFRvIII receptor and GFP were labeled with a biotinylated-antibody against EGFRvIII followed by streaptavidin-Alexa647 followed biotin-NLP-RPE, and analyzed by fluorescence microscopy for both Alexa647 and RPE. (**b**) Gli36 cells were infected with lentivirus vector expressing BAP-TM and GFP. These cells were labeled with Streptavidin-Alexa647 followed by biotin-NLP-RPE and analyzed as in (**a**).

To determine the functionality of the newly synthesized biotin-NLP-RPE liposome in targeting tumor cells, viable Gli36 human glioma cells overexpressing both the mutant EGFRvIII, a frequent genetic alteration in primary brain tumors^21^ and GFP, or plain Gli36 control cells were incubated with a biotinylated antibody against EGFRvIII followed by Streptavidin-Alexa647 and finally by biotin-NLP-RPE and analyzed by fluorescence microscopy. A distinct surface staining of both Alexa647 (indicative of streptavidin binding) and rhodamine-PE (liposome) was observed, showing the functionality of this liposome in targeting tumor cells (Fig. 4a). The plain Gli36 cells did not show any surface staining (data not shown).

To evaluate the usefulness of the biotin-NLP-RPE in targeting tumor cells in a different model, Gli36 human glioma cells were engineered by a lentivirus vector to express both GFP (marker for transduction efficiency) as well as a fusion protein between a biotin acceptor peptide (BAP), preceded by a signal sequence, and the transmembrane domain (TM) of the PDGFR receptor (BAP-TM)^22^. Upon gene transfer and expression, the biotin ligase will tag the BAP peptide with a single biotin moiety which is presented on the cell surface through TM. These cells as well as control cells were then labeled with streptavidin-Alexa647 followed by incubation with biotin-NLP-RPE and analyzed by fluorescence microscopy. A distinct cell surface staining was found on cells expressing BAP-TM for both Alexa647 (streptavidin biotin) and RPE (liposome binding) proving the specific targeting of biotin-NLP-RPE (Fig. 4b). Control cells did not show any surface staining (data not shown).

### Targeted multimodal *in vivo* imaging of tumors using biotin-NLP-RPE

**Figure 5:**
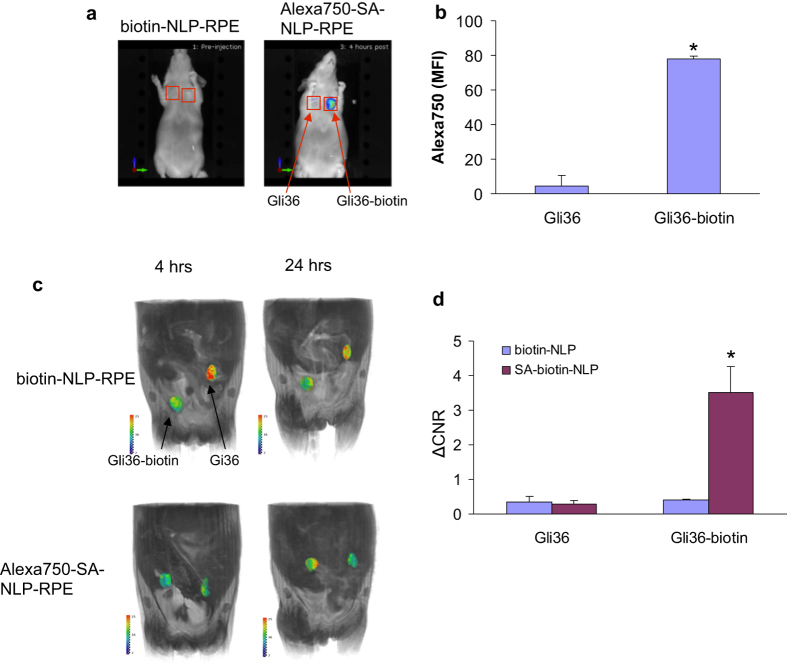
Targeting and *in vivo* imaging of tumors with biotin-NLP-RPE. Gli36 human glioma cells or same cells expressing biotin on their surfaces (through BAP-TM) were implanted subcutaneously in nude mice (n = 5) at 2 different sites. Two-weeks later, mice were i.v. injected with either biotin-NLP-RPE or a complex consisting of biotin-NLP-RPE and streptavidin-Alexa750 (Alexa750-SA-NLP-RPE) and imaged 4 hrs later with FMT Alexa750. (**a**,**b**) A representative mouse from each group is shown in (**a**) and quantitation of fluorescent accumulation in each tumor in (**b**). (**c**,**d**) The same mice were also imaged with T1w MRI at 4 and 24 hrs post-injection (**c**). T1w intensities at 24 hrs were normalized to 4 hrs for each tumor (**d**). *p < 0.001 as calculated by student’s t-test.

To confirm the functionality of biotin-NLP-RPE in an *in vivo* model, Gli36 cells engineered by gene transfer to express BAP-TM or plain Gli36 control cells were injected subcutaneously in the flanks of nude mice (n = 5) at two different locations. Two-weeks later, mice were i.v. injected with either biotin-NLP-RPE or a complex consisting of biotin-NLP-RPE and streptavidin-Alexa750 (Alexa750-SA-NLP-RPE) and imaged first with fluorescence-mediated tomography (FMT; 4 hrs post-injection) for Alexa750 confirming streptavidin targeting. Significant accumulation (*p < 0.001) of Alexa750 signal was observed in tumors expressing biotin on their surfaces as compared to control tumors in mice injected with Alexa750-SA-NLP-RPE and not the non-targeted biotin-NLP-RPE (without the use of streptavidin), showing efficient targeting of this NLP complex to biotin expressing tumors (Fig. 5a,b). The same mice were also imaged with T1w MRI at 4 and 24 hrs post-injection. Significantly higher accumulation (*p < 0.001) of NLP liposome over time was observed in tumors expressing biotin on their surfaces as compared to control tumors only in mice injected with targeted Alexa750-SA-NLP-RPE complex showing that tumors can be targeted and imaged with MR using this liposome complex (Fig. 5c,d). Mice injected with non-targeted biotin-NLP-RPE liposome showed similar and lower accumulation in both tumors.

## Discussion

In this study, we synthesized a new liposome and characterized it by comparing its efficacy as a contrast agent to a more conventional liposome. We showed that the new liposome NLP exhibited four-fold higher longitudinal and five-fold higher transversal relaxivity compared to a conventional liposome CLP. Further, the longitudinal relaxivity (at 1.41 Tesla) of NLP was >2-fold higher compared to a published liposome formulation with the same Gd-DOTA containing lipid (29.9 mM^−1^s^−1^ compared to 12.8 mM^−1^s^−1^)^20^. We showed that the NLP formulation is thermosensitive and we observed a temperature dependency for both longitudinal and transversal relaxivities being lower at higher temperature. Recent studies showed that an increase in temperature leads to an increase in longitudinal relaxivity for DSPC-based Gd(III)DOTA-DSPE containing liposomes (12.8 mM^−1^s^−1^ at 37 °C compared to over 14 mM^−1^s^−1^ at 67 °C)^20^. A potential explanation for these differences between typical liposomes and NLP could be the increased water exchange across the liposomal membrane at higher temperature resulting in larger contribution of Gd(III)DOTA-DSPE in the inner leaflet of the liposome to the overall longitudinal relaxivity^20^,^23^,^24^,^25^. The unique composition of the NLP liposome could possibly positively affect the water exchange across the liposomal membrane and explain its high relaxivities compared to both CLP and Gd(III)DOTA-DSPE containing liposome. To further increase the sensitivity and selectivity of the NLP contrast agent, and to make it useful for multimodal imaging, we synthesized the NLP liposome with biotin on its surface and rhodamine within the lipid bilayer. The Gd(III) inside the liposome can serve as a contrast agent for MRI and the rhodamine can be used for imaging at the single cell level with fluorescence. This biotin-NLP-RPE showed to have three to four-fold higher relaxivities compared to CLP. Further, we showed that streptavidin can serve as a bridge between biotin-NLP-RPE and biotinylated antibody to target specific receptors overexpressed on tumor cells.

*In vivo* optical imaging has limited use due to the problem of light absorption by pigmented molecules such as hemoglobin and scattering by mammalian tissues, leading to a decrease in the detection limit. The biotin-NLP-RPE was synthesized with a rhodamine-containing lipid in the bilayer, which was shown to be useful in targeting tumor cells at the single cell level in culture using fluorescence imaging. This same complex would be useful for single cell detection *in vivo* using intravital micoscopy and could be extended for intraoperative fluorescence imaging^26^. Further, the rhodamine can be replaced with any fluorophore emitting in the near infra-red region of the spectrum, which can then be used for FMT imaging in deep tissues *in vivo*^27^. The biotin on the NLP surface makes these liposomes universal targeted contrast agents for which practically any peptide/antibody specific to a cell of interest can be complexed to it through the strong interaction of biotin with streptavidin. The specificity of biotin to streptavidin has been exploited for several medical and scientific applications including drug or toxin targeting, *in vivo* imaging of targeted cells, and antibody-guided radioimmunotherapy in humans^22^,^28^,^29^.

The use of biotin-NLP-RPE liposome can be extended and applied for drug delivery. The combination of imaging with drug delivery is very valuable in the search for new therapeutic strategies. Further, the thermosensitive characteristic of this liposome does not only have a positive effect on the relaxivity, but could also be used as a delivery strategy for drugs, for instance to solid tumors. Our *in vitro* data shows that a temperature of 39 °C can lead to an increase in calcein release from NLP liposomes. This thermosensitive feature of NLP has a high clinical impact in which NLP liposome can encapsulate a therapeutic drug which can be released specifically on the tumor site by ultrasound-induced hyperthermia, similar to previously published methods^10^,^15^,^16^. Further, by including Gd(III) in these liposomes, MRI can be used for guided drug release at the specific site^10^.

In conclusion, we have synthesized a new liposome with higher r1 and r2 relaxivities as compared to conventional liposome and showed it to be useful for targeting and multimodal imaging of tumors. Since this liposome is thermosensitive, it can be used for ultrasound-mediated drug delivery at specific sites, such as tumors, which can be guided by MRI or catheter based optical imaging^30^,^31^,^32^,^33^.

## Methods

### Materials for liposome preparation

1,2-Dipalmitoyl-sn-glycero-3-phosphocholine (DPPC), 1,2-ditetradecanoyl-*sn*-glycero-3-phosphocholine (DMPC), 1,2-distearoyl-sn-glycero-3-phosphoethanolamine-N-[methoxy(polyethylene glycol)-2000] (PEG2000-DSPE), 1,2-distearoyl-sn-glycero-3-phosphoethanolamine-N-[biotinyl(polyethylene glycol)2000] (Biotin-PEG2000-DSPE), 1,2-Dioleoyl-sn-Glycero-3-Phosphoethanolamine-N-(Lissamine Rhodamine B Sulfonyl) (Rhodamine PE), 1,2-distearoyl-sn-glycero-3-phosphoethanolamine-N-[maleimide(polyethylene glycol)-2000] (PEG2000-DSPEmal), 1,2-Distearoyl-sn-glycero-3-phosphocholine (DSPC) and cholesterol were purchased from Avanti Polar Lipids (Alabaster, AL). Gd(III)DOTA-DSPE and Gd(III)-DTPA-bis(stearylamide) (Gd(III)-DTPA-BSA) were obtained from Gateway Chemical Technology (St. Louis, MO).

### Preparation of liposome formulations

Liposomes were prepared according to three different formulations: the more conventional liposome DSPC-Gd(III)DTPA-BSA (CLP), the new formulation DPPC-Gd(III)DOTA (NLP) and the new formulation biotinylated and rhodamine-labeled: DPPC-Gd(III)DOTA-biotin (biotin-NLP-RPE). NLP liposome was prepared with DPPC, DMPC, Gd(III)DOTA-DSPE and PEG2000-DSPE at a molar ratio of 50:20:25:5 (Table 1). As a control, CLP liposome were used containing DSPC, Gd(III)DTPA-BSA, PEG2000-DSPE, PEG 2000-DSPEmal and cholesterol in a molar ratio of 36.7: 25:2.5:2.5:33.3 (Table 1). The biotin-NLP-RPE liposome was prepared with DPPC, DMPC, Gd (III)DOTA-DSPE, PEG2000-DSPE, Biotin-PEG2000-DSPE and Rhodamine PE at a molar ratio of 50:20:25:2.5:2.5:0.1 (Table 1). These liposomes were prepared by lipid film hydration followed by extrusion^5^. The lipid film was prepared by dissolving the lipid mixture (50 μmol) in 3:1 v/v CHCl_3_/MeOH in a 250 ml round-bottomed flask. The solution was evaporated using rotation evaporation for 15 minutes at 200 mbar and 30 °C and at 150 mbar till dryness. In order to assure complete dryness, rotation evaporation was pursued at 0 mbar for 15 more minutes and the film was subsequently put under a nitrogen flow for at least one hour. The NLP and biotin-NLP-RPE films were dried overnight. The films were hydrated with 4 ml HEPES buffered saline (HBS) (20 mM HEPES and 135 mM NaCl at pH 7.4) at 60 °C for NLP and biotin-NLP-RPE and 65 °C for the CLP liposomes after heating the film to 65 °C. The films of the NLP and biotin-NLP-RPE liposomes were not heated before addition of the HBS. The lipid dispersions were extruded (Lipofast Extruder, Avestin, Ottawa, Ontario) at 60 °C and 65 °C for the NLP and CLP liposomes respectively. The lipid dispersions were extruded through polycarbonate membrane filters (Whatman, Maidstone, UK) with a pore diameter of 400 nm (1 time), through filters with a pore diameter of 200 nm (6 times) and through filters with a pore diameter of 100 nm (6 times).

### Liposome characterization

The size and size distribution of liposomes were measured using dynamic light scattering (DLS) at 25, 37 or 41 °C on a Malvern Zetasizer Nano S apparatus (Malvern, UK) equipped with a 633 nm laser. These measurements were performed in sextuple using three different concentrations of liposome suspension in 400 μl HBS (20 mM HEPES and 135 mM NaCl at pH 7.4).

Total lipid and gadolinium [Gd(III)] concentrations of liposome suspensions were calculated using phosphate determination, performed according to Rouser after destruction of the samples with perchloric acid at 180 °C^34^. The amount of phospholipids in the sample was determined using a calibration curve between 0 and 80 nmol phosphate. The total amount of lipids and the amount of gadolinium containing lipids in the sample was calculated based on the measured amount of phospholipids.

The gadolinium ion concentration was determined for NLP and biotin-NLP-RPE liposomes using inductively coupled plasma mass spectroscopy (ICP-MS; DRCII Perkin-Elmer, Philips Research Material Analysis, Eindhoven, The Netherlands). From each sample, 150 μl was taken, weighed and sent in for ICP (supplementary data).

### Determination of thermosensitivity

The thermosensitivity of NLP liposome was determined based on calcein release experiment and differential scanning calorimetry. In order to measure calcein release, NLP liposome films (25 μmol) were hydrated with 2 ml of 30 mM calcein 100 mM NaCl solution in HBS (pH 6.8). Extrusion was performed according to the same protocol as for the NLP liposome. Free calcein was separated from calcein-containing liposome using PD-10 column (GE-healthcare). Liposome was then dissolved in HBS (20 mM HEPES and 135 mM NaCl; pH 7.4). The fluorescent intensity of the calcein-containing liposome was measured with a fluorometer (excitation wavelength: 494 nm; emission wavelength: 517 nm). As a control, the fluorescent intensity of the liposome was measured at room temperature before and after addition of 10% triton-X in water. The fluorescent intensity was measured every minute for 40 minutes. The first measurement was performed at 25 °C and the temperature of the water bath increased by 1 °C every minute. The temperature of the sample was measured every two minutes. After 40 minutes, 10% triton-X in water was added to the sample to determine the maximal fluorescent intensity. The phase transition temperature of NLP liposome was measured with differential scanning calorimetry (DSC, Universal V4 5A TA Instruments).

### Determination of relaxivity

To determine the longitudinal and transversal relaxivities of the liposomes, spin-lattice relaxation time (T1) and spin-spin relaxation time (T2) measurements were performed on a Bruker (Rheinstetten, Germany) Minispec mq60 NMR analyzer (60 MHz) with a magnetic field of 1.41 Tesla. T1 relaxation times were obtained using the standard inversion recovery method with a recycle delay of 20 s, inversion time ranging from 5 ms to 10 seconds, the inversion sequence was repeated ten times and four averages. T2 times were measured using a Carr-Purcell-Meiboom-Gill (CPMG) sequence with a recycle delay of 20 s, interecho time of 0.4 ms, 10,000 echoes and 16 averages.

The longitudinal r1 and the transversal r2 relaxivities were obtained from the slope of the linear fit of relaxation rates as a function of gadolinium ion concentration described in equation 1 and 2 respectively (below). The relaxation rates R1 and R2 are the inverse of T1 and T2 respectively. The longitudinal and transversal relaxivities are a measure for the efficacy of the contrast agent.


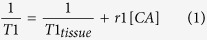



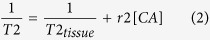


[CA] is the concentration of the contrast agent, in this case the Gd(III) concentration in mM and r1 and r2 the longitudinal and transversal relaxivity respectively^35^. In order to determine the relaxivities of the CLP, NLP and biotin-NLP-RPE liposomes, T1 and T2 measurements were performed at 37 °C. Since NLP liposome is thermosensitive, relaxivities were determined at different temperatures. T1 and T2 measurements were performed within a temperature range of 22 °C to 67 °C with 5 °C intervals.

### Cell culture

Gli36 human glioma cells were obtained from Dr. Anthony Capanogni, UCLA, CA. Gli36 cells were transduced with a retrovirus vector expressing the mutant EGFRvIII and puromycin resistant gene (obtained from Dr. Miguel Sena-Esteves, Department of Neurology, University of Massachusetts Medical Center, Worcester). The cells were selected by culturing the cells in conditioned media with 1 mg/l puromycin. Gli36 cells were also infected with a lentivirus vector expressing a biotin acceptor peptide, preceded by a signal sequence followed by the transmembrane domain of PDGFR (BAP-TM) as previously described^22^. As a control, Gli36 cells were infected with similar empty lentivirus vector. All cells were cultured in Dulbecco’s modified Eagle’s medium (DMEM) supplemented with 10% fetal bovine serum (Sigma, St. Louis, MO) and 100 U penicillin and 0.1 mg streptomycin (Sigma) per milliliter at 37 °C in a 5% CO_2_ humidified incubator.

### *In vitro* targeting of glioma cells with biotin-NLP-RPE

Seventy five thousand cells were plated in 1.5 ml conditioned media on coverslips (Fisher Scientific) in a 24 well plate. Twenty-four hours later, cells were incubated with 250 ng/ml of biotinylated anti-EGFRvIIII antibody for 5 min (in phosphate buffer saline, PBS, a kind gift from Dr. Darell D. Bigner, Duke University Medical Center) at room temperature. Cells were then washed and incubated with streptavidin-Alexa647 (1:200; Molecular Probes) for 5 min. Cells were washed again and incubated with biotin-NLP-RPE (70 μM) for 5 min. Cells were then washed, fixed with 4% paraformaldehyde, mounted on coverslips and analyzed by fluorescence microscopy. For cells expressing BAP-TM, similar protocol was followed without the use of the antibody.

### Targeting and Multimodal imaging of tumors with biotin-NLP-RPE *in vivo*

All animal studies were approved by the Subcommittee on Research Animal Care at Massachusetts General Hospital and were performed in accordance to their guidelines and regulations. Mice were kept on biotin-deficient diet throughout the study. One million Gli36 cells (in 50 μl) expressing BAP-TM or control cells were mixed with similar volume of Matrigel (Becton Dickinson) and implanted subcutaneously in the flanks of nude mice in 2 different locations. Two weeks later, mice (n = 5) were injected with a complex of streptavidin-Alexa750 (200 μg) and biotin-NLP-RPE (200 μl of 7 mM) pre-incubated for 15 min. Mice were then imaged with fluorescence-mediated tomography and magnetic resonance at different time points.

### Magnetic resonance imaging

MRI was performed using an animal 4.7 T MRI scanner (Bruker, Billerica, MA) consisting of coronal and axial T1w images (rapid acquisition with refocused echoes (RARE) sequence, TR = 900 ms, TE = 14.1 ms, field of view = 5.4 cm x 4.0 cm, matrix size = 256 × 256, slice thickness = 1 mm, 16–18 slices) obtained before and after intravenous administration of Alexa750-SA-NLP-RPE or biotin-NLP-RPE as control as above. Post contrast images were obtained at 4 and 24 hrs post injection. Image analysis and segmentation was performed using the OsiriX™ DICOM viewer. Contrast-to-noise ratios were computed as (ROI_tumor_ – ROI_muscle_)/STD(noise), where ROI = region of interest drawn to encompass the tumor on the slice where the tumors are the largest or a region of flank muscle, and STD = standard deviation. For the 3D MRI image, the tumors were segmented out manually in the Amira environment (Visage Imaging, Inc., San Diego, CA). This was volume rendered in pseudocolor. The rest of the body was volume rendered in gray scale.

### Fluorescence-mediated tomography

Quantitative fluorescent tomographic imaging was carried out on a commercial imaging system (FMT2500, Perkin Elmer, Waltham MA). Prior to imaging, mice received an intravenous injection of imaging agent. Four hours after injection, mice were non-invasively imaged under general isoflurane anesthesia (1–1.5% at 2l/min). Paired absorption and fluorescent data sets were collected using a scanning laser, and 3-dimensional reconstructions were generated utilizing the TrueQuant FMT software.

## Additional Information

**How to cite this article**: Kuijten, M. M. P. *et al.* Multimodal targeted high relaxivity thermosensitive liposome for *in vivo* imaging. *Sci. Rep.* **5**, 17220; doi: 10.1038/srep17220 (2015).

## Figures and Tables

**Table 1:** Formulation of CLP, NLP and biotin-NLP-RPE liposomes.

CLP	Mol %	NLP	Mol %	NLP-biotin-RPE	Mol %
DSPC	36.67	DPPC	50	DPPC	50
Gd(III)DTPA-BSA	25	DMPC	20	DMPC	20
Cholesterol	33.33	Gd(III)-DOTA-DSPE	25	Gd(III)-DOTA-DSPE	25
PEG2000-DSPE	2.5	PEG2000-DSPE	5	PEG2000-DSPE	2.5
PEG2000-DSPEmal	2.5			Biotin-PEG2000-DSPE	2.5
				Rhodamine PE	0.1

**Table 2:** Characterization of CLP, NLP and biotin-NLP-RPE liposomess.

Characteristics	CLP	NLP	NLP-biotin-RPE
Size	109 nm	86 nm	93 nm
Liposome concentration	11.16 mM	7.70 mM	7.0 mM
Concentration Gd(III) by phosphate determination	2.790 mM	1.925 mM	1.75 mM
Concentration Gd(III) by ICP	—	2.238 mM	1.91 mM

**Table 3:** Longitudinal (r1) and transversal (r2) relaxivities in mM^−1^s^−1^of CLP, NLP and biotin-NLP-RPE liposome formulations at 37 °C obtained from NMR measurements at 60 MHz at 1.5 Tesla.

Relaxivity at 37 °C	CLP	NLP	NLP-biotin-RPE
Relaxivity r1	7.52 mM^−1^s^−1^	29.88 mM^−1^s^−1^	23.86 mM^−1^s^−1^
Relaxivity r2	10.64 mM^−1^s^−1^	49.02 mM^−1^s^−1^	39.02 mM^−1^s^−1^
r2/r1	1.41	1.64	1.64
